# Immunotherapy with allotumour mRNA-transfected dendritic cells in androgen-resistant prostate cancer patients

**DOI:** 10.1038/sj.bjc.6602761

**Published:** 2005-08-30

**Authors:** L J Mu, J A Kyte, G Kvalheim, S Aamdal, S Dueland, M Hauser, H Hammerstad, H Waehre, N Raabe, G Gaudernack

**Affiliations:** 1 Section for Immunotherapy, The Norwegian Radium Hospital, University of Oslo, Montebello, Oslo 0310, Norway; 2 Laboratory of Cellular Therapy, The Norwegian Radium Hospital, University of Oslo, Montebello, Oslo 0310, Norway; 3 Department of Clinical Cancer Research, The Norwegian Radium Hospital, University of Oslo, Montebello, Oslo 0310, Norway; 4 Department of Radiology, The Norwegian Radium Hospital, University of Oslo, Montebello, Oslo 0310, Norway; 5 Department of Surgery, The Norwegian Radium Hospital, University of Oslo, Montebello, Oslo 0310, Norway; 6 Department of Oncology, The Norwegian Radium Hospital, University of Oslo, Montebello, Oslo 0310, Norway

**Keywords:** dendritic cell, immunotherapy, prostate cancer

## Abstract

Here, we present results from a clinical trial employing a new vaccination method using dendritic cells (DCs) transfected with mRNA from allogeneic prostate cancer cell lines (DU145, LNCaP and PC-3). In all, 20 patients were enrolled and 19 have completed vaccination. Each patient received at least four weekly injections with 2 × 10^7^ transfected DCs either intranodally or intradermally. Safety and feasibility of vaccination were determined. Immune responses were measured as delayed-type hypersensitivity and by *in vitro* immunoassays including ELISPOT and T-cell proliferation in pre- and postvaccination peripheral blood samples. Serum prostate-specific antigen (PSA) levels and bone scans were monitored. No toxicity or serious adverse events related to vaccinations were observed. A total of 12 patients developed a specific immune response to tumour mRNA-transfected DCs. In total, 13 patients showed a decrease in log slope PSA. This effect was strengthened by booster vaccinations. Clinical outcome was significantly related to immune responses (*n*=19, *P*=0.002, *r*=0.68). Vaccination with mRNA-transfected DCs is safe and results in cellular immune responses specific for antigens encoded by mRNA derived from the prostate cancer cell lines. The observation that in some patients vaccination affected the PSA level suggests that this approach may become useful as a treatment modality for prostate cancer patients.

Prostate cancer is one of the most common types of cancer in Western countries, and is the second leading cause of cancer death in men after lung cancer. Patients with metastatic disease usually receive palliative hormone treatment. Still, most of these patients will eventually develop hormone-refractory disease with increasing prostate-specific antigen (PSA) levels. Cancer vaccines may represent an alternative form of therapy for such patients. It has been shown that autologous dendritic cells (DCs) pulsed with peptides specific for PSA (Heiser *et al*, 2000), prostate-specific membrane antigen (PSMA) (Tjoa *et al*, 1996) or telomerase reverse transcriptase (TERT) (Vonderheide *et al*, 1999) are capable of stimulating potent CTL responses *in vitro* and data from clinical trials are now available (Heiser *et al*, 2002; Kaufman *et al*, 2004). Since prostate cancer cells are genetically unstable and as such represent ‘shifting targets’ (Shaffer and Scher, 2003), single-agent vaccines may result in selection of genetic variants that escape the immune attack. The use of vaccines containing multiple tumour-derived antigens may elicit a broader antitumour response than single antigen vaccines and circumvent to some extent the problem of immune escape (Heiser *et al*, 2001).

Several strategies for whole tumour vaccines are available. Fusion between autologous tumour cells and allogenic DCs has recently been used as semiallogeneic tumour vaccines in clinical trials (Gong *et al*, 2000; Trefzer *et al*, 2000). For hormone-resistant prostate cancer patients with increasing PSA, tumour cells are generally not available for this approach. An alternative to autologous tumour material would thus be the use of allogeneic prostate cancer cell lines. In a rat model, the prophylactic use of allogeneic tumour vaccination resulted in tumour protection of the animals (Hrouda *et al*, 2000). A similar approach has recently been used in humans (Eaton *et al*, 2002). Dendritic cells pulsed with lysates of allogeneic prostate cancer cell lines has also been tested in a phase I/II clinical study (Pandha *et al*, 2004). Recently, we have developed a clinical scale method for GMP (good manufacturing practice) production of allogeneic mRNA-transfected DC as an alternative approach (Mu *et al*, 2003). We here present data from our first clinical study, indicating that the treatment is safe and results in specific immune responses as well as decrease of PSA level in a majority of the patients.

## MATERIALS AND METHODS

### Patient selection

Inclusion criteria were histologically verified adenocarcinoma of the prostate with evidence of disease progression as assessed by increasing PSA in three subsequent analyses while on LHRH agonist, or following orchiectomy with or without antiandrogen medication. All patients had their antihormonal treatment continued through the vaccination period and 3 months following the therapy. Age >45 years with ECOG performance score <2, adequate haematologic, renal and hepatic function was required (details not shown). Prior radiotherapy and chemotherapy must have elapsed a minimum of 4 weeks prior to entry in the study. Patients with a history of disease not suitable to vaccines were excluded. Patients in the two arms of this study (intradermal (i.d.) or intranodal (i.n.) injection) were included on a consecutive basis and were not stratified. The trial was approved by the Norwegian Medicines Agency, the Norwegian Department of Health Gene Therapy Board and the Regional Committee for Medical Research Ethics, and performed in compliance with the Helsinki declaration. Written informed consent was obtained from all patients.

### Preparation of mRNA-transfected DCs

Monocyte-derived DCs were generated as described previously (Mu *et al*, 2003). Briefly, peripheral blood mononuclear cells (PBMC) obtained by leukopheresis were enriched for monocytes by immunomagnetic depletion of B cells and T cells using ISOLEX 300i magnetic cell selector (Nexell, Irvine, CA, USA). After 5 days culture in Teflon bags, using serum-free CellGro DC medium (CELLGenix, Freiburg, Germany) supplemented with granulocyte/macrophage colony-stimulating factor (2500 U ml^−1^) and interleukin-4 (IL-4, 1000 U ml^−1^) (CELLGenix), immature DCs were generated. Bulk tumour mRNA was extracted from three human prostate cancer cell lines DU145, LNCaP and PC-3 (obtained from ATCC, Manassas, VA, USA) corresponding to 5 × 10^7^ cells of each type for each vaccine preparation and mixed before transfection. A square-wave electroporation procedure was employed to transfect mRNA (120 *μ*l, 0.2–1 *μ*g *μ*l^−1^) into immature DCs. Quality of all RNA preparations were controlled by electrophoresis on agarose gels stained by Gel star (Cambrex Bio Science, Verviers, Belgium). Selected mRNA preparations were also evaluated on an Agilent Bioanalyser instrument using RNA 6000 Nano Reagent & Supplies (Agilent Technologies, Palo Alto, CA, USA). The Bioanalyser quantifies RNA by calculating size and concentration of each separated band (Kyte *et al*, 2005). Transfection efficiency was monitored in separate experiments with enhanced green fluorescent protein mRNA instead of tumour mRNA as described (Mu *et al*, 2003). Cells were matured for 48 h, using the same medium supplemented with IL-1*β* (10 ng ml^−1^), IL-6 (1000 U ml^−1^), tumour necrosis factor *α* (10 ng ml^−1^) (CELLGenix) and prostaglandin E_2_ (1 *μ*g ml^−1^) (Sigma-Aldrich, USA). Mature DCs were then frozen in aliquots containing 2 × 10^7^ vial^−1^ and stored in liquid nitrogen until use. Mock-transfected DCs were cultured following the same electroporation procedure without mRNA and frozen as described above. Cell quality including phenotype, viability and sterility were monitored for each step.

### DC vaccination

Frozen mRNA-transfected DCs were thawed in a 37°C water bath, washed once and resuspended in 200 *μ*l PBS for vaccination. Each patient received at least four weekly injections employing 2 × 10^7^ DCs each time. Of the 20 patients being enrolled, 10 of them had the DCs injected by ultrasound guidance directly into the paracortex of an inguinal lymph node (i.n.), while the others received the vaccine i.d.

### Evaluation of immune responses

**Figure 1:**
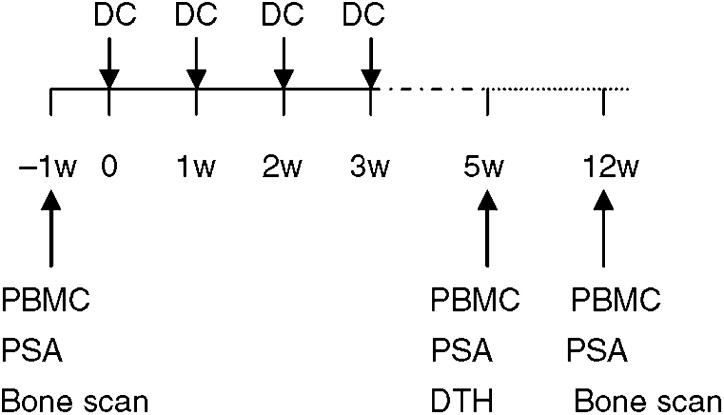
Flow chart of trial.

Blood samples were taken from patients before vaccination (baseline), 5 weeks and 3 months after the start of vaccination (Figure 1). PBMC were isolated and frozen in liquid nitrogen until testing.

#### T-cell proliferation test

The T-cell-enriched nonadherent fraction of PBMC was obtained by a plastic adherence step and used as responder cells. Irradiated (30 Gy) tumour mRNA-transfected DCs (DCt) and mock-transfected DCs (DCn) were used as stimulators. Responder cells 5 × 10^4^ well^−1^ were cultured in triplicate with various numbers of stimulators for 5 days at 37°C, 5% CO_2_ in CellGro DC medium. ^3^H-thymidine (Laborel, Oslo, Norway) was added 18 h before testing. Data were reported as counts per minute (c.p.m.). Medium only, responder cells only and stimulator cells only were used as negative controls.

#### ELISPOT assay

The same responder and stimulator cells as described above were used. Dendritic cells as stimulators were plated 4 × 10^3^ well^−1^, while the responder cells were added in a titration from 1 × 10^5^ well^−1^ to 1.25 × 10^4^ well^−1^ in duplicate. Details have been described previously (Mu *et al*, 2003). Spots per 10^5^ T cells were calculated. Results were recorded positive when the mean number of spots in the DCt wells differed significantly from that of the DCn.

#### Generation of T-cell clones and specificity test

T-cell clones were generated from responder cells that gave a positive proliferation response. Cells were incubated in CellGro DC medium by limiting dilution (0.3/1/3 cells well^−1^). Irradiated (30 Gy) allogeneic PBMC (10^6^ ml^−1^) were used as feeder cells, and 1 *μ*g ml^−1^ PHA and 20 U ml^−1^ IL-2 were added. Clones obtained were tested by proliferation test described above and also by a standard ^51^Cr cytotoxicity test (Heiser *et al*, 2001). For cytotoxicity assay, DCt, DCn and prostate cancer cell lines DU145, LNCaP and PC-3, respectively, were used as targets.

#### Delayed-type hypersensitivity (DTH) reaction

Tumour mRNA-transfected DCs and DCn were administered to patient i.d. in week 5 at separate sites. The diameters of the erythema were measured and recorded by the patient 48 h after injection. Since skin reactions also regularly developed towards the DCn, it was accordingly scored as positive when the erythema diameter of DCt was ⩾10 mm and also at least 5 mm bigger than that of the DCn.

### Clinical monitoring

#### Assessment of safety

Adverse events were evaluated and graded according to the standard criteria (NCI-CTC criteria version 2.0). The relation between adverse events and treatment was also given as probable, suspected, unlikely or not related to treatment. Adverse events were considered as being related to the treatment if the relationship was reported as probable or suspected.

#### Assessment of clinical response

Clinical progression was defined as the appearance of new lesions of malignant disease, or the development of symptoms consistent with metastatic disease. Response was also assessed as PSA levels in serum. PSA measurements were carried out before therapy, at week 5 and month 3 after the start of vaccination. A partial PSA response was defined as a reduction of PSA by greater than 50% from baseline, stable disease was defined as a reduction of less than 50% or an increase of less than 50%, and progressive disease was defined as an increase of greater than 50% (Kaufman *et al*, 2004).

### Statistical analysis

The pre- and postvaccination data were assessed by two-way ANOVA (SPSS). The linear regression model was used to obtain estimates of the change in serum PSA over time. Statistical significance was determined at *P*< 0.05. All statistical tests were two-sided.

## RESULTS

### Patient characteristics

In this study, DCs were prepared from 20 patients. Of these, 19 patients completed four vaccinations, while one discontinued due to tumour progression before vaccination was initiated. The characteristics of the 19 patients are summarised in Table 1. All patients had hormone-resistant disease with increasing PSA levels and 14 patients had bone metastases at the time of inclusion. At the start of treatment, five patients had anaemia grade I and four patients had hypoalbuminaemia grade I. One patient had serum aspartate aminotransferase (AST) and alanine aminotransferase (ALT) levels grade I and another patient had serum creatinine level grade I. The rest of the patients had haemoglobin, albumin, AST, ALT and creatinine within normal levels. All patients had bilirubin within normal levels as well as total number of white cells, neutrophils and platelets above lower normal limits. All the patients with anaemia and or hypoalbuminaemia had positive bone scans at the start of treatment.

### Safety of DC therapy

In this study, 10 patients received i.n. vaccinations, while the other nine patients received i.d. injections. At least four vaccine doses were administrated to each patient. Five patients also received boost injection (P08, P10, P16, P17 and P19). Vaccination was well tolerated with no grade II–IV toxicity. Minor symptoms related to the vaccination treatment were erythema and increased size of groin lymph nodes at the injection site, minor pain at the injection site or a small increase in hot flushes. These symptoms were seen in only a few of the patients and were considered negligible. Between the end of vaccination and evaluation at the 3rd month after the start of treatment, one patient started Taxotere treatment at another clinic. Blood tests showed no treatment-related neutropenia, thrombocytopenia, renal or liver toxicity. All the six patients with anaemia at the 3rd month had positive bone scans.

### Production and quality testing of mRNA-transfected DCs

After B- and T-cell depletion, blood samples contained a mean value of 53.1%. (27–65%) CD14+ cells, 1.3% (0–10%) B cells and 5.4% (1–9%) T cells. Following culture procedure including electroporation, the produced cells showed mature DC morphology and phenotype characterised by no or very low proportion of CD14, but high expression of CD86 and HLA-DR with variable expression of CD83 (Table 2). Thawing and washing of the frozen samples gave a mean viability of 86.6% (56–98%), with a mean cell yield of 8.2% (1.9–19.6%) of the total PBMC used.

### T-cell monitoring and functional studies

**Figure 2:**
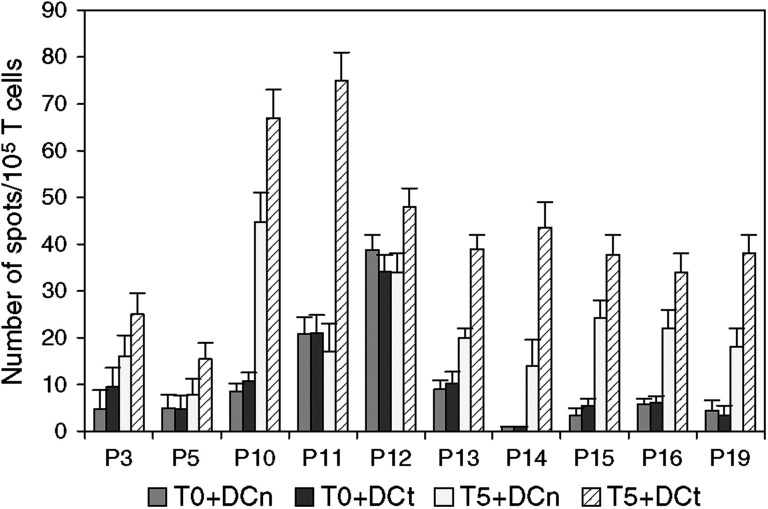
ELISPOT result in the10 patients with positive response. Data show INF*γ* spots per 10^5^ T cells. T0: T cells from before vaccine; T5: T cells from the 5th week after vaccine. DCt: tumour mRNA-transfected DC; DCn: mock-transfected DC. Results are recorded as mean number of INF*γ* spots with 95% confidential interval (CI) indicated on the bars.

**Figure 3:**
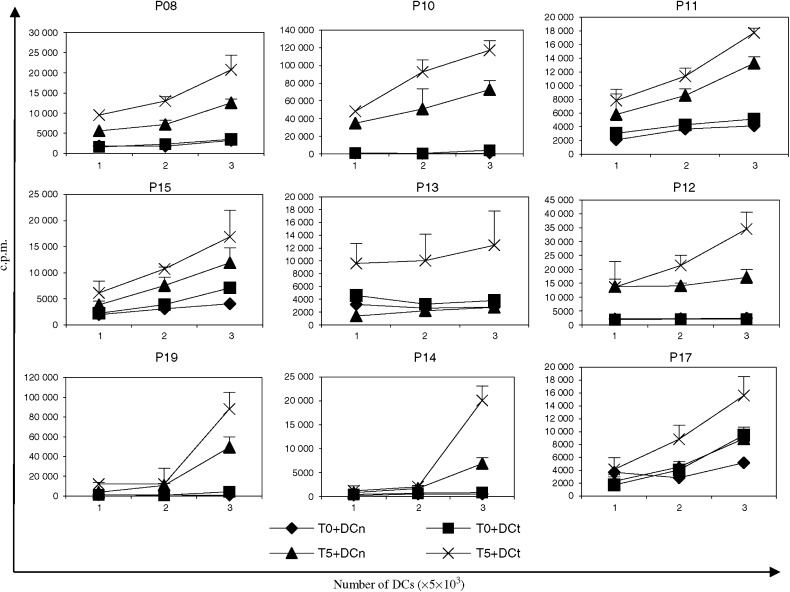
T-cell proliferation test from the nine patients with positive result. T0: T cells from before vaccine; T5: T cells from the 5th week after vaccine. DCt: tumour mRNA-transfected DC; DCn: mock-transfected DC. Results are recorded as mean c.p.m. of triplicate with 95% confidential interval (CI) indicated on the bars.

Of 19 patients treated, 10 showed positive response in the ELISPOT test. Result recorded in Figure 2 represents number of spots per 10^5^ T cells in the assay. The mean frequency of specific T cells after vaccination was calculated to be 18.8/10^5^ T cells (range 6–50/10^5^ T cells), which represents a 14-fold increase compared with prevaccine samples. Of the responding patients, three were given i.n. administration and seven were injected i.d. Following vaccination, T cells from most patients also showed stronger response to DCn than what was found prior to vaccination, suggesting that a considerable autologous mixed lymphocyte reaction (MLR) was generated during the vaccination. Even with this MLR component obscuring the specific response, a significant proliferation response specific for DCt was observed in nine patients (Figure 3), seven of these were also ELISPOT positive. Compared to T cells from prevaccine samples, postvaccine T cells showed a clear dose-dependent response with increasing numbers of DCs well^−1^. The emergence of both an MLR response as well as specific T-cell response following vaccination is evident. A decline in T-cell proliferation was usually observed over the next 3 months compared to the responses on week 5, but the immune response could be boosted by the administration of additional vaccine injections (data not shown).

**Figure 4:**
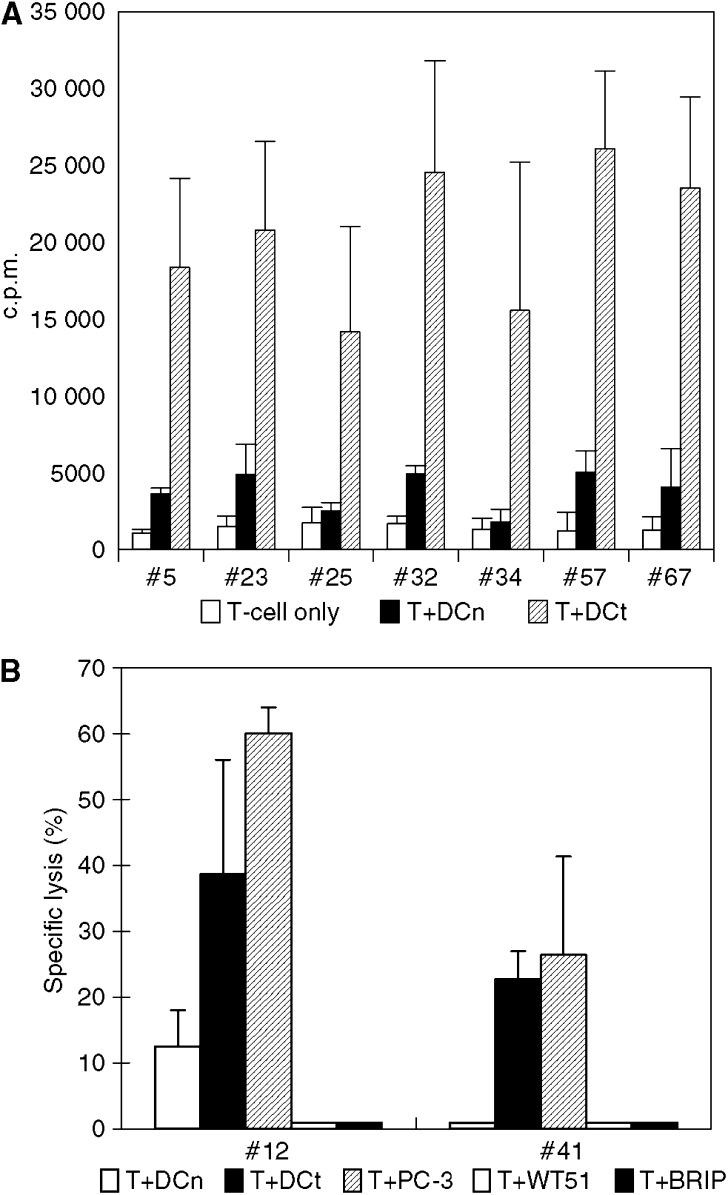
(**A**) Proliferative responses of Th clones from patient 19. In all, 72 T-cell clones were generated from postvaccine T cells, of these 10 were specific for transfected DC. All were of the CD4+ phenotype. Seven representative positive clones are shown here. Results are given as mean c.p.m. of triplicate wells with standard error of the mean (s.e.m.) indicated on the bars. (**B**) Killing of ^51^Cr target cells by CTL clones derived from patient 10. Two of CD8+ clones were derived from P10, who shared HLA-A9 with the tumour cell line PC-3. Results are given as specific lysis of target cells. The prostate cancer cell line PC-3 and mRNA-transfected DCs are killed, but no lysis when HLA-matched EB cell lines were used as targets. The ratio of effector: target in this experiment was 25 : 1. The results are recorded as mean lysis of triplicate with s.e.m. indicated on the bars.

T-cell clones were generated from patients with a positive response. Figure 4A shows results of proliferation test of T-cell clones derived from P19. These clones generated from postvaccine T cells were considered specific for antigens encoded by tumour–mRNA since they responded significantly to DCt. All of the clones were CD4+ confirmed by flow cytometry. From patient 10, 46 T-cell clones were generated, two of which were CD8+. Patient 10 shared the HLA-A9 antigen with the tumour cell line PC-3. We accordingly tested if these potential CTL clones would recognise this cell line in a standard cytotoxicity assay using DCn and DCt as negative and positive controls. HLA-A9-matched Epstein–Barr virus (EBV)-transformed B-cell lines (WT51 (HLA-A23) and BRIP (HLA-A24)) from the 10th IHWS homozygous cell panel were also used as control target. The results depicted in Figure 4B demonstrate that these CTL clones specifically killed DCt and more efficiently also the PC-3 cell line, but did not lyse EBV cells. In separate experiments, no killing of the two other cell lines used for preparation of mRNA was observed (data not shown). Taken together, these results demonstrate that vaccination with DCt gives rise to a broad T-cell response including both the CD4+ and the CD8+ T-cell subsets.

### Delayed-type hypersensitivity

Five of the 19 tested patients were positive in the DTH test (Table 2). For three patients, DTH result was not available. Median erythema diameter of the DTH-positive patients was 17 mm (10–62 mm). Considerable DTH reactivity against DCn was also developed during vaccination, necessitating a stringent definition of a positive response. This probably made the results of the DTH response less informative.

### Clinical response

Although the primary goal of this phase I/II trial was to assess safety and immune responses to the DC vaccine, patients were also assessed for early clinical response registered 3 months after entry into the vaccine trial (Table 2).

**Figure 5:**
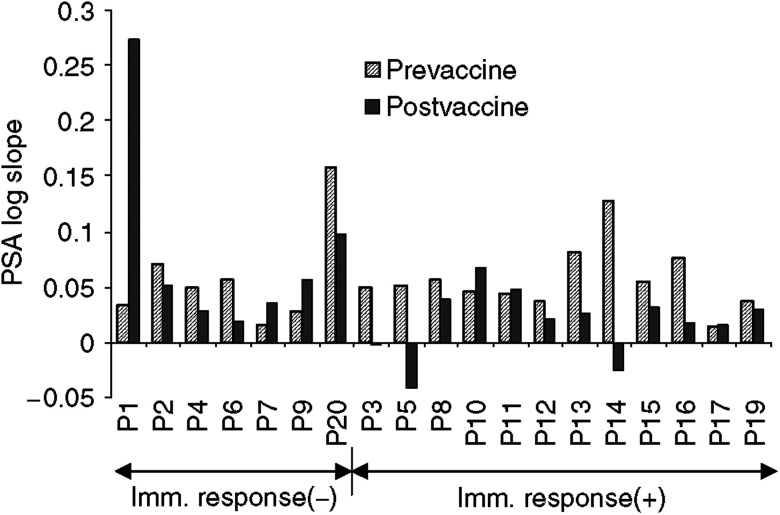
Log slope PSA values for the whole group of patients. Postvaccine PSA was evaluated by the end of the 3rd month after vaccination. Four of seven immune response-negative patients and nine of 12 immune response-positive patients showed decrease in slope.

**Figure 6:**
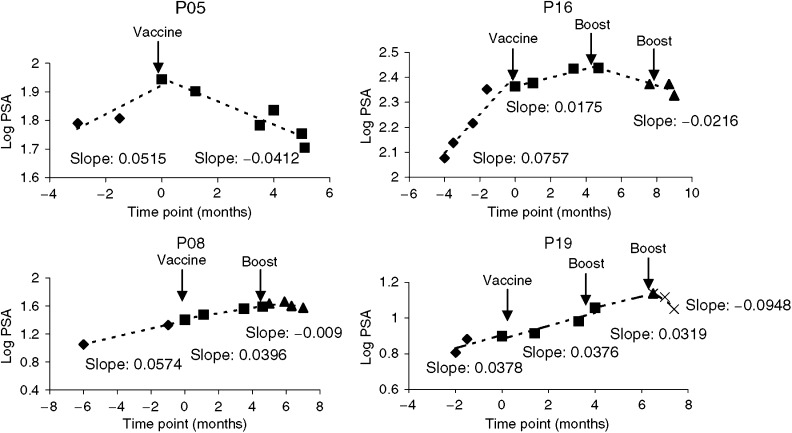
A linear regression model used to estimate change of serum PSA over time. Four representatives were shown here. P05 showed decrease in PSA value immediately after vaccination. The other three patients showed gradually decrease in slope but more clearly after boost. PSA value began to decrease following one additional boost in P08 and P16 and two additional boosts in P19.

No improvement in bone scan was observed. However, patients with positive bone scan prior to therapy disclosing immunological response had unchanged bone scans 3 months after therapy. By PSA response criteria, 11 patients fell into the criteria of stable disease. Six patients (P02, P03, P05, P11, P13, and P14) showed a 0.6–48.7% decrease of serum PSA value. Although the absolute PSA value continued to rise in most of the patients, a decrease of log slope PSA (PSA velocity) after vaccination was noted in 13 patients out of 19 patients (Figure 5). Figure 6 shows log PSA measurements at different time points in four representative patients, among whom three were given one to two additional boost injection(s). Before vaccination, all patients had a log-linear rising serum PSA. Following vaccination, the rising slope decreased (P16), kept stable (P08, P19) or became negative (P05). In the three patients given boost injection(s), the log slope PSA decreased further indicating that biochemical response comes late and that such patients may need to be boosted for a longer time period. Among the 11 patients with evidence of disease stabilisation on the 3rd month, 10 had developed specific T-cell response, while only two of eight patients with disease progression showed T-cell response. Thus, a clear correlation between immune response and early clinical response was established (*n*=19, *P=*0.002, *r*=0.68).

## DISCUSSION

The present study describes the results of a phase I/II clinical study of a vaccine based on allogeneic tumour mRNA transfected autologous DC in advanced prostate cancer. There is growing evidence indicating that loading a broad spectrum of antigens rather than single antigens onto DCs potentially may give rise to more efficient anticancer immune responses (Nencioni *et al*, 2003; Kalady *et al*, 2004). In the present study, we used mRNA isolated from three human prostate cancer cell lines instead of primary tumour from individual patient as a source of polyvalent prostate cancer antigens. This approach was chosen due to lack of sufficient biopsy material and has some disadvantages. The two most important being that certain individual antigens present in the patient's own tumour may be absent from the vaccine and that the vaccine contains numerous irrelevant antigens, such as allo-HLA antigens and minor antigens encoded by a broad array of polymorphisms. The absence of important individual antigens is a general flaw in the majority of vaccine approaches, including all of the molecularly defined vaccines except those that represent antigens encoded by mutations such as K-Ras mutations (Gjertsen *et al*, 1997). We believe that this aspect to some extent has been taken care of in this study by combining several different prostate cancer cell lines. The prostate cancer cell lines used were selected so as to include one cell line (LNCaP) secreting a known prostate cancer antigen PSA and the two other that represent androgen-resistant cancer. The presence of allo-HLA antigens has been used as nonspecific antigens in other approaches (Marten *et al*, 2003), and may enhance the response rather than interfering negatively. However, on the other hand, such T-cell responses may obscure the specific responses and make interpretation of the T-cell data more difficult.

We here document that sufficient number of vaccine quality DCs could be generated successfully in all 20 patients enrolled. Vaccination was well tolerated by the administration of both i.d. and i.n. routes. No systemic toxicity was observed. Therefore, using tumour cell line mRNA-transfected DCs as cancer vaccine is safe.

Although the results from the *in vitro* T-cell tests and the *in vivo* DTH reactions did not completely correlate to each other, the main observation were very similar. Specific T-cell responses were obtained in more than half of the patients. The nature of the immune response was not studied in detail in this clinical study, but in a preclinical evaluation, we analysed a broad range of cytokines using the Bioplex system. These results demonstrated that a broad spectrum of cytokines was produced in the *in vitro*-generated T-cell response (LJ Mu *et al*, unpublished; Kyte *et al*, 2005) All of the patients who mounted a specific T-cell response also developed a component of MLR reactive cells recognising DCn. This also held true for most of the patients who failed to raise a specific T-cell component. This response was clearly vaccine dependent since very little reactivity was observed in prevaccine samples. Based on a review of the results from clinical DC vaccine trials published, this finding was unexpected, but similar results have recently been published in a non-small-cell lung cancer DC vaccine trial (Hirschowitz *et al*, 2004). Evidence for some prevaccine T cells response specific for DCt was also observed in three patients (Figures 2 and 3), indicating that a cellular immune response to some prostate cancer antigens might be induced spontaneously during the disease process. Reactivity against known candidate prostate cancer antigens such as PSA will be tested in an attempt to characterise the response in more detail.

In patients with progressive prostate cancer, the rise in serum PSA is associated with an exponential growth rate (log-linear) (Schmid *et al*, 1993; Patel *et al*, 1997). This was also seen in all our patients at study entry. Of six patients with PSA higher than 100 *μ*g l^−1^ at entry, there were only two developing immune response to the vaccine. Of the 13 patients with PSA lower than 100 *μ*g l^−1^ at entry, 10 showed positive immune response. Patients with lower PSA tended to give a higher level of response. In this study, it appears that i.d. injections gave better T-cell responses (eight of nine) than those given i.n. administration (four of 10). However since the i.d. group had lower median PSA (18.9 *μ*g l^−1^) than the i.n. group (median 112.2 *μ*g l^−1^), we cannot presently conclude that one route of injection is better than the other. Using level of PSA as a surrogate marker for clinical response, 13 patients demonstrated a decrease in PSA log slope. In some patients, changes in PSA were only observed after boost injection(s), indicating that repeated vaccinations over a longer period might be required for clinical efficacy. Similar results were recently also reported in a clinical study involving prostate cancer patients vaccinated with hTERT mRNA-transfected DCs (Su *et al*, 2005). Since one component of the mRNA used in this trial is encoding PSA, it may be argued that a change in log slope PSA may result from the induction of PSA-specific antibodies by the vaccine. We did not measure PSA antibodies in the serum of our patients, but others using different forms of vaccines against PSA have failed to induce such antibodies (Barrou *et al*, 2004; Kaufman *et al*, 2004). In light of the high average PSA level in the patients in the present study, we find it unlikely that the amount of PSA produced by DCt would contribute much to the total PSA level in serum and hence the stimulation of B cells in the patients.

In prostate cancer patients, DCs loaded with different types of antigens have been used as vaccines. These antigens include recombinant mouse prostate-acid phosphatase (PAP) (Fong *et al*, 2001), human PAP (Small *et al*, 2000), PSA proteins or RNA (Heiser *et al*, 2002; Barrou *et al*, 2004), PSMA peptides (Tjoa *et al*, 1996), allogeneic tumour lysate (Pandha *et al*, 2004) and RNA amplified from autotumour cells (Heiser *et al*, 2001). In general, DC vaccination turns out to be safe to patients and evidence for clinical benefit is accumulating. Comparisons of clinical effects among the different trials are difficult since the clinical stages of the patients studied are not the same in the individual protocols. The approach followed by Pandha *et al* is very similar to the one used by us, with the major difference that cell lysates rather than mRNA was used as a source of antigen. Thus, they used two of the three cell lines included in our protocol (LNCaP and DU 145). In their study, they observed a similar frequency of immune responders but a somewhat smaller clinical response, as only three of the nine patients showed a fall in PSA or reduction in PSA velocity. The latter may be the result of giving lower numbers of DCs and fewer injections, as some of the patients in our study only showed an effect on PSA velocity after booster vaccinations. Although the number of patients in both studies is small, the results of the two studies point in the same direction, indicating that the use of a panel of allogeneic prostate cancer cells may be represent an alternative to autologous tumour vaccines. To our knowledge, this is the first report of a clinical trial using mRNA from different cancer cell lines, representing a broad repertoire of prostate cancer antigens. The correlation between T-cell responses and a favourable early clinical outcome encourages further studies involving patients in earlier stage of the disease. It would also be of interest to perform a two-arm study comparing the efficacy of DCs loaded with either tumour cell lines mRNA or lysate from the same cell lines.

In order to further improve the clinical effect of our vaccination approach, several issues needs to be addressed. Since some patients had clinical responses only after booster vaccination, this indicates that longer treatment periods may be needed to get full effect of the vaccine. To intensify the immune response, shorter intervals between each boost vaccination may also have effect. Furthermore, it has been shown that less than 1% DCs injected i.d. can migrate to secondary lymph nodes (Morse *et al*, 1999). This means that the ‘active dose’ of the vaccine in our case is less than 2 × 10^5^ DCs. Clearly, there is room for much improvement here. Since the function and the maturation stage of DCs can influence the vaccine results greatly (Jonuleit *et al*, 2001), a definite establishment of the DC phenotype that correlates with good clinical responses is needed. More recent trials in our institution concentrate on using Toll-like receptor 7 agonists (Imiquimod) applied to the skin to enhance the migration of DCs, and enhancement of the T-cell responses by infusion of cytokines, for example, IL-2 in combination with the DC vaccines.

## Figures and Tables

**Table 1:** Patient characteristics

**Characteristics**	**Value**
Age (years) (median, range)	69 (48–78)

*Stage of disease when diagnosed* a	
T2	4
T3	15

Gleason score (median, range)	8 (7–9)
Interval between diagnosis and vaccination (median, range)^b^	56 (19–153)
Baseline PSA (median, range)^c^	50 (7.9–2571)
Bone scan positive before vaccine	14

PSA=prostate-specific antigen.

a Two of them were N1M0, 12 were N0M0 and five were NxMx.

b Months.

c Microgram per litre.

**Table 2:** Vaccine characteristics and results

**Patient ID**	**Vaccine route**	**Quality of DC %CD83/%CD86/ %HLA-DR**	**Number of vaccinations**	**DTH**	**Immune response**	**Log PSA slope^a^**	**Clinical outcome**
P01	i.n.	27.8/98.7/95.2	4	−	−	−	PD^b^
P02	i.n.	25.7/92.6/81.7	4	NT	−	+	SD^b^
P03	i.n.	34.0/95.8/89.3	4	−	+	+	SD
P04	i.n.	13.1/96.1/88.3	4	−	−	+	PD^b^
P05	i.n.	44.7/97.7/91.9	4	+	+	+	SD^b^
P06	i.n.	11.1/94.3/93.6	4	+	−	+	PD^b^
P07	i.n.	91.4/94.4/96.1	4	NT	−	−	PD^b^
P08	i.n.	91.0/99.7/99.8	5	+	+	+	SD^b^
P09	i.n.	97.0/99.7/98.6	4	NT	−	−	PD^b^
P10	i.d.	94.6/99.5/97.8	5	+	+	−	PD^b^
P11	i.d.	72.7/92.5/88.1	4	−	+	−	SD^b^
P12	i.d.	92.0/99.0/96.3	4	−	+	+	SD^b^
P13	i.d.	86.0/97.0/89.7	4	−	+	+	SD
P14	i.d.	90.5/99.0/96.6	4	+	+	+	PD^b^
P15	i.d.	95.7/98.8/99.3	4	−	+	+	SD^b^
P16	i.d.	94.8/98.8/98.4	6	−	+	+	SD
P17	i.d.	84.8/99.6/97.3	5	−	+	+	SD
P19	i.n.	90.6/99.4/97.0	6	−	+	+	SD^b^
P20	i.d.	96.1/98.5/97.2	4	−	−	−	PD

DC=dendritic cells; DTH=delayed-type hypersensitivity; PSA=prostate-specific antigen; i.n.=intranodal; i.d.=intradermal; NT=not tested; SD=stable disease; PD=progressive disease.

a Decrease in slope marked as ‘+’.

b Indicate the patient is positive in bone scan.
